# Unfolded Protein Responses With or Without Unfolded Proteins?

**DOI:** 10.3390/cells1040926

**Published:** 2012-11-01

**Authors:** Erik L. Snapp

**Affiliations:** Department of Anatomy and Structural Biology, Albert Einstein College of Medicine, 1300 Morris Park Avenue, Bronx, NY 10461, USA; Email: erik-lee.snapp@einstein.yu.edu; Tel.: +718-430-2967; Fax: +718-430-8996

**Keywords:** unfolded protein response, endoplasmic reticulum, misfolded protein, BIP, stress, Ire1, inositol

## Abstract

The endoplasmic reticulum (ER) is the site of secretory protein biogenesis. The ER quality control (QC) machinery, including chaperones, ensures the correct folding of secretory proteins. Mutant proteins and environmental stresses can overwhelm the available QC machinery. To prevent and resolve accumulation of misfolded secretory proteins in the ER, cells have evolved integral membrane sensors that orchestrate the Unfolded Protein Response (UPR). The sensors, Ire1p in yeast and IRE1, ATF6, and PERK in metazoans, bind the luminal ER chaperone BiP during homeostasis. As unfolded secretory proteins accumulate in the ER lumen, BiP releases, and the sensors activate. The mechanisms of activation and attenuation of the UPR sensors have exhibited unexpected complexity. A growing body of data supports a model in which Ire1p, and potentially IRE1, directly bind unfolded proteins as part of the activation process. However, evidence for an unfolded protein-independent mechanism has recently emerged, suggesting that UPR can be activated by multiple modes. Importantly, dysregulation of the UPR has been linked to human diseases including Type II diabetes, heart disease, and cancer. The existence of alternative regulatory pathways for UPR sensors raises the exciting possibility for the development of new classes of therapeutics for these medically important proteins.

## 1. Introduction

**Figure 1:**
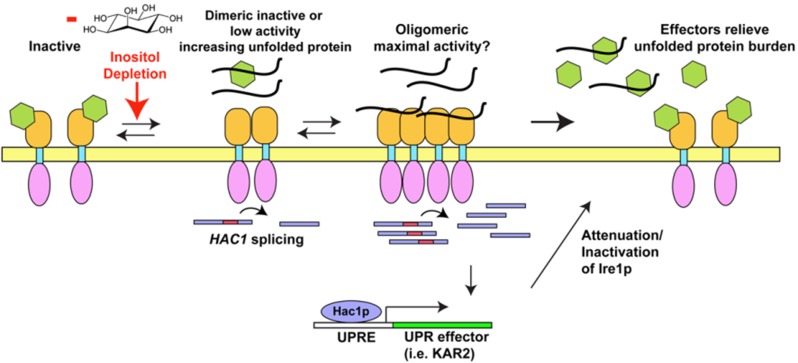
Illustration of Ire1p activation and inactivation. Unfolded protein accumulation and inositol depletion stimulate BiP/Kar2p release and Ire1p oligomerization. Splicing of *HAC1* mRNA is further enhanced by peptide binding to Ire1p and Ire1p clustering. Increased levels of Hac1p upregulate UPR effectors leading to resolution of stress.

Nascent secretory proteins enter the endoplasmic reticulum (ER) lumen and immediately encounter a myriad of quality control (QC) effectors that enhance correct protein folding and prevent and resolve accumulation of misfolded secretory proteins in the ER [1,2]. The QC effectors include chaperones, folding enzymes, and ER-associated degradation (ERAD) retrotranslocation machinery [3]. The effectors are constitutively expressed, but can be dramatically upregulated by the integral membrane sensors that mediate the Unfolded Protein Response (UPR) [4]. The sensors include Ire1p (in the yeast *S. cerevisiae* [5]) and IRE1α (highly homologous to the yeast Ire1p), PERK, and ATF6) [6,7,8,9] in metazoans. Each UPR stress sensor has a discrete set of downstream targets that help a cell resolve an unfolded protein burden [3,4,10,11]. PERK transiently attenuates global protein translation to decrease the nascent protein burden [12]. In addition, ER volume expands to help dilute any accumulation of unfolded proteins in the ER [13]. All of the sensors are integral membrane proteins. Upon unfolded protein accumulation, the sensors activate by either oligomerizing or, in the case of ATF6, exit the ER through vesicular trafficking to the Golgi complex for proteolytic processing. PERK and IRE1α /Ire1p each form homo-oligomers and stimulate their effectors, *i.e.*, *HAC1* mRNA splicing for Ire1p (Figure 1) [6,14]. PERK phosphorylates eIF2α to attenuate general translation [12]. IRE1α cleaves *XBP1* (Ire1p similarly cleaves *HAC1*) mRNA and a tRNA ligase splices the transcription factor in frame, enabling the correctly spliced form to upregulate chaperones and ERAD components [6,15]. ATF6 is proteolytically cleaved, releasing a transcription factor [6,16]. Similar to Xbp1 and Hac1p, the ATF6 transcription factor upregulates ER QC machinery [17]. If the metazoan UPR fails to resolve the stress, caspases can be activated leading to cell death [18]. For this reason, UPR resolution is considered critical for a cell survival. Curiously, in yeast, which lacks a metazoan apoptotic pathway, it also appears to be critical to keep the UPR below a certain threshold for survival [19,20]. As several human diseases have been linked to insufficient or excessive UPR [21,22,23], understanding the fundamental mechanisms of the UPR is a medically important goal. While much is known concerning the biology of the effectors, the activation and attenuation of the UPR sensors remain less well understood. In the following review, we will focus on the prototypical ER stress sensor, Ire1p and its human homolog IRE1α and recent progress on these problems.

## 2. Conceptual Model for Sensing Stress

Before analyzing the specific case of Ire1p/IRE1α regulation, I'd like to briefly consider the general problem of sensing and resolving stressful conditions. Cells have evolved several different strategies for detecting and responding to environmental conditions that perturb homeostasis, thus causing forms of "stress". There are two fundamental steps for stress pathways: (1) Detection of the stressful condition and (2) activation of a cellular protein or network of proteins to enact a program that enables the cell to adapt and/or restore homeostasis. The potential solutions for these requirements are numerous. A stressful substrate could inhibit, activate or alter the stability of sensor or effector proteins. Once activated, the sensor or effector could undergo further regulation by covalent posttranslational modifications, such as phosphorylation

In simple cases, a stressor is a unique metabolite that directly binds a sensor. However, many stresses are broadly acting physico-chemical changes, *i.e.*, heat, osmotic or oxidative damage, that impact a broad array of targets. The sensing of misfolded protein accumulation falls into this more challenging category, in that proteins can misfold in numerous ways and few proteins share conserved sequences. Cellular chaperone proteins generally can recognize a diverse variety of incompletely folded or misfolded proteins often through exposure of hydrophobic domains that are usually buried in a properly folded protein [24]. Chaperones assist in protein folding and prevent protein aggregation. Thus, a chaperone or a chaperone domain would be a logical solution for sensing a misfolded protein accumulation.

One of the earliest misfolded protein stress pathways is the bacterial heat shock response that involves a transcription factor, σ32, that is negatively regulated by the bacterial Hsp70, DnaK [25]. σ32 has a short half-life and can be inactivated by binding to DnaK. Upon stress, unfolded protein levels overwhelm DnaK, titrating available pools of DnaK from σ32, leading to increased levels and stability of σ32, which, in turn, stimulates transcription of DnaK and other chaperones whose genes have σ32 elements in their promoters [25]. In eukaryotes, a similar strategy is employed for the Heat Shock Response. The cytoplasmic Hsp90 ATPase chaperone binds the Heat Shock Factor 1 (HSF1) transcription factor [26]. During nonstressful conditions, a small fraction of the highly expressed pool of Hsp90 molecules binds HSF1 and retains the protein in the cytoplasm. Stresses that increase levels of misfolded cytoplasmic proteins increase occupancy of the main pool of Hsp90 with clients. Liberation of Hsp90 permits HSF1 to trimerize, enter the nucleus, and increase transcription of *HSP70*, *HSP90*, and other chaperones and effectors to resolve the misfolded protein stress [26]. Attenuation of the Heat Shock Response is not well understood, but requires inactivation of HSF1, which appears to involve acetylation of HSF1 [26].

In the ER, the secretory protein folding environment also contains several chaperones and must be protected from accumulating misfolded proteins. However, sensing of accumulating misfolded secretory proteins must be transmitted across a membrane barrier. One solution for the sensing problem would be to evolve a protein that detects misfolded proteins and one or more transmembrane proteins to convey the signal across the ER membrane. The sensor and effector could even be part of a single integral membrane protein sensor/effector molecule. Once activated, the response could be attenuated by either degrading the sensor protein, post-translational modifications to the sensor and/or some obstruction of the ability to interact with effector molecules. The integral ER membrane protein, Ire1p in yeast and IRE1α in mammalian cells, satisfies at least some of these criteria and is essential for regulating the mRNA for the key UPR effector, *HAC1* in yeast and *Xbp1* in metazoans [4].

## 3. Ire1p and IRE1α Structure and Function

The yeast enzyme Ire1p was first identified in a screen for inositol auxotrophs, leading to the name Inositol Requiring Enzyme (Ire1p) [27]. *IRE1* was essential for yeast to grow on synthetic media lacking inositol. The following year, the team of Kazu Mori and Mary-Jane Gething and the team of Peter Walter and Jeff Cox both reported that yeast lacking *IRE1* could not grow on stressors that induced the accumulation of misfolded secretory proteins, including the compounds tunicamycin (Tm) and DTT, which prevented N-linked glycosylation of N-X-S/T consensus peptides or disrupted disulfide bond formation, respectively [28,29]. The metazoan UPR is sensitive to the sarco/endoplasmic reticulum Ca^2+^ ATPase (SERCA) calcium channel inhibitor thapsigargin (Tg), which depletes the ER of calcium [30]. Potential clues to the mechanism of Ire1p function came from the recognition that the Ire1p cytoplasmic domain contained significant homology to the mammalian ribonuclease RNase L [31] and the observation that the mRNA of the genetically linked transcription factor, Hac1p, was spliced during ER stress only in cells that expressed *IRE1* [29], though splicing did not significantly increase Hac1p stability [47]. Ire1p performs the specific cytoplasmic RNA cleavage event, while a tRNA ligase completes the splicing reaction [32]. The metazoan IRE1 similarly cleaves the *Xbp1* transcription factor mRNA [33].

### 3.1. Ire1p/IRE1α Structure

Ire1p and IRE1α are single pass Type I integral membrane proteins [27]. The yeast protein has four potential N-linked glycosylation sites while the human form has one. Both proteins have three luminal cysteines. Neither the N-linked glycosylation sites nor the cysteines appear to influence Ire1p activity [34]. Kenji Kohno's lab has extensively mutagenized Ire1p and characterized five functionally distinct subdomains of the NH_2_-terminal luminal domain [35]. In particular, Domains II-IV were defined as the "core stress-sensing" domains, with Domain IV being especially important for dimerization of the luminal domain during stress and Ire1p activation [34]. Domain V was identified as the Kar2p binding domain [35] (the ER Hsp70, BiP in mammalian cells, see Section 5. Domains II and IV, when dimerized, form a structure capable of binding unfolded peptides [36,37,38]. On the cytoplasmic side, the COOH-terminal domain contains a kinase domain and an RNase domain [27,28,29]. The RNase domain performs at least three effector roles including: *HAC1*/*Xbp1* mRNA cleavage, degradation of ER membrane associated mRNAs (termed Regulated Ire1-Dependent Decay (RIDD)), and 28S ribosomal cleavage [33,39,40,41,42,43]. Metazoan IRE1 has been frequently considered a pro-survival arm of the UPR [44,45]. However, the overexpressed form of IRE1α used to arrive at this conclusion was kinase dead and not capable of the pro-apoptotic RIDD activity in wt IRE1α [42]. Thus, at different stages in the stress response, IRE1α can be either protective or pro-death. While many of the mechanistic details of Ire1p/IRE1α structure-function have been determined, the initial steps of activation and the mechanism of attenuation of Ire1p/IRE1α remain enigmatic.

### 3.2. Ire1p Functional Assays

Ire1p undergoes changes in oligomerization, phosphorylation, clustering, and *HAC1* RNA splicing activity. The spliced *HAC1* output can be measured by multiple assays including Northern blot of spliced mRNA, RT PCR of spliced and unspliced mRNA, fluorescent signal from the product of an out of translational reading frame fluorescent protein mRNA that Ire1p splices into frame, changes in transcript levels of a Hac1p transcription factor targeted gene (*i.e.*, *INO1* or *KAR2*), measurement of fluorescence or β-galactosidase activity produced by a transcriptional reporter containing a UPR response element promoter upstream of a fluorescent protein or LacZ reporter, assays of Ire1p clustering (FRET, live cell fluorescence imaging, and immunofluorescence) and growth of yeast on plates under stressful conditions. As astutely noted in Credle *et al.* [38], the choice of assay can have very different outcomes, as the outputs will depend on the stability of the product and whether amplification of the product occurs during the assay. For example, a single transcription factor could theoretically produce many transcripts, which in turn could produce multiple proteins from each transcript—a significant amplification of signal. Similarly, fluorescent proteins tend to be highly stable, where as spliced *HAC1* mRNA has a short half-life (~20 min) [46] and Hac1p has a half-life of two min [47,48]. Measurement of spliced *HAC1* levels is one of the few assays that permit detection of inactivation of Ire1p following stress resolution. Pincus *et al.* cleverly found that changes in the *rate* of GFP synthesis could serve as a comparable surrogate measure [49]. Several of the assays report features increasingly removed from the specific actions of Ire1p. For example, spliced *HAC1* levels are a combined measure of amount of spliced mRNA and mRNA stability. Importantly, unspliced *HAC1* is relatively stable, which will affect ratiometric quantitation [50]. In light of these caveats, comparisons of Ire1p activity suggest that minor differences in Ire1p splicing activity can result in dramatic differences in downstream UPR activity. When considering the effects of various mutations on Ire1p function, the choice of assay and timing of the readout can impact interpretation of the consequences of the mutation.

## 4. Ire1p/IRE1α Activation Models

What are the mechanistic steps required for Ire1p/IRE1α activation? A wide range of stimuli- pharmacologic, mutant misfolded proteins, and manipulations that alter luminal ER chaperone availability for nascent proteins- have been correlated with an Unfolded Protein Response [11] and specifically with Ire1p/IRE1α activation [51]. Ire1p activation includes oligomerization and phosphorylation [14,52] and correlates temporally at very early times with the accumulation of unfolded secretory proteins. Similarly, increasing occupancy of the ER Hsp70 chaperone BiP with unfolded secretory protein clients is coincident with the earliest downstream events of *Xbp1* splicing [53]. Misfolded secretory protein levels could be sensed by Ire1p/IRE1α (1) directly by Ire1p/IRE1α, (2) through Ire1p/IRE1 α regulation by BiP, and/or (3) by additional proteins.

### 4.1. Density Dependent Activation of Ire1p

Different studies suggest Ire1p/IREα needs to be restrained from activating. Overexpression of IRE1α/Ire1p activates constitutive *Xbp1*/*HAC1* splicing, though not as robustly as a Tm stress [52,54]. Constitutive overexpression of IRE1α is not tolerated by mammalian cells, presumably due to the apoptotic consequences of RIDD [42]. In the absence of regulatory factors, the purified Ire1p cytoplasmic domain dimerizes *in vitro* independent of the luminal domain [55,56] or phosphorylation [20,55], but appears to require binding to ADP [20]. The purified luminal domain also readily dimerizes [36,57]. Thus, as concentrations of Ire1p/IRE1α increase, the proteins become increasingly prone to collisions, leading to oligomerization, a requirement for activation. These findings suggest that Ire1p/IRE1α could be activated simply by density dependent collisions or that overexpression in cells could titrate an inhibitory factor. Increased levels of Ire1p/IRE1α could also increase the probability of encountering a hypothetical activating factor. There is no evidence that Ire1p/IRE1α expression levels change significantly prior to or during UPR activation. In fact, Ire1p is normally expressed at exceptionally low levels in most cell types. Given the propensity of purified Ire1p domains to oligomerize *in vitro*, the need for one or more inhibitory molecules to regulate Ire1p is logical.

### 4.2. Ire1p Sensing of Unfolded Proteins

Ire1p/IRE1α oligomerizes as unfolded secretory proteins accumulate in the ER [14,52]. Oligomerization is essential for Ire1p/IRE1α function [38,58,59]. Monomeric Ire1p does not undergo phosphorylation or perform *HAC1* splicing [58]. Oligomerization is a prerequisite for phosphorylation [52]. Crystal structures have been solved for both the luminal [38,60] and cytoplasmic domains [56,61,62,63] of Ire1p and IRE1α. Interestingly, the purified domains crystallize as a pair of twisting strands. Each strand is assembled from oligomers of repeating dimers. The monomers bind each other at two distinct interfaces to enable formation of higher order oligomers [38,56]. Whether the oligomers that form in cells are highly ordered Ire1p/IRE1α complexes is unclear. Nor is it obvious how many copies of Ire1p/IRE1α are in cellular oligomers or what the functional minimal oligomer is. However, evidence from Kimata *et al.*, Li *et al.* and Aragón *et al.* reveals that Ire1p and IRE1α both cluster in the ER membrane during stress [37,54,58]. Transmission electron micrographs suggest the clusters even cause localized distortions in the ER membrane [37]. Studies of mutagenized dimerization interfaces of Ire1p found higher order clustering was required for maximal activation and Ire1p splicing activity [36,37,58].

The UPR can be activated to differing degrees over a large range of compound concentrations [10,49]. Even outputs (upregulated genes) can differ for stresses that quantifiably activate Ire1p to comparable levels [64]. Thus, the UPR is not an all or nothing system. Ire1p/IRE1α appears to be able to detect differences between stresses and respond proportionately. This might happen through modulation of a negative regulatory factor or even through direct detection of changes in unfolded secretory protein levels.

**Figure 2:**
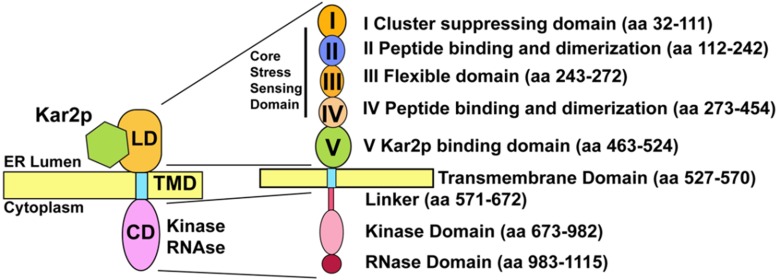
Functional domains of the Ire1p. LD = Luminal Domain, TMD = transmembrane domain, CD = cytoplasmic domain.

A growing body of data support direct detection of unfolded secretory proteins by Ire1p [36,37,65]. On the basis of the structural similarity of the Ire1p to the peptide-binding groove of MHC-1, Peter Walter proposed the peptide-binding hypothesis. In this model, Ire1p directly senses the accumulation of unfolded secretory proteins in the ER lumen by binding the peptides to the MHC-1-like domain. The Ire1p luminal domain (Subdomains II–IV) (Figure 2), upon dimerization, can demonstrably bind unfolded protein domains [36,37,38]. Mutagenesis of key residues in the major histocompatibility complex 1 (MHC1)-like peptide binding cleft decreases Ire1p mutant activation during misfolded secretory protein accumulation [36,38]. The ability of Ire1p to bind unfolded proteins correlates reasonably well with the ability to acutely stimulate Ire1p phosphorylation and *HAC1* splicing activity. Binding unfolded peptides is important for higher order oligomerization [37,58]. Aragón *et al.,* reported that peptide binding stimulated Ire1p clustering, maximized *HAC1* recruitment to Ire1p, leading to maximal *HAC1* splicing [58]. Kimata *et al.*, [37] reported that binding to the peptide-binding domain stimulated reorientation of the cytoplasmic domain to maximize splicing activity. A follow-up study by Gardner and Walter revealed that growth on Tm was poor for a yeast mutant (MFY) with decreased affinity for unfolded secretory peptides (see Supplemental Online Materials Figure S7 in [36]). Together, these observations satisfy the criteria to define the sensing function of a stress pathway.

There are caveats to the peptide-binding model. To bind peptides, it seems likely Ire1p would need to be in a minimally dimeric form. Gradient fractionations and co-IPs suggest this is not the case in homeostatic cells [14,66]. Gardner and Walter [36] reported detecting little evidence of monomer binding to peptide. Also, in the Gardner study, the ΔMFY mutations decreased the affinity for a substrate ~50-fold, but only decreased splicing reporter signaling by one half. Similarly, cells expressing a mutant Ire1p lacking a functional peptide binding domain (ΔIII) still splice *HAC1* mRNA during DTT or Tm-induced stress, but much less acutely, taking three h instead of 15–60 min to achieve maximal splicing [59]. The MFY mutant cells in the Gardner study still grew, albeit poorly, unlike cells lacking *IRE1*, which failed to grow. 

The peptide binding story is further complicated in humans. The MHC1-like domain is too narrow to interact with peptides in Randall Kaufman's group's crystal structure of the human IRE1α luminal domain [60]. They also reported that the putative peptide binding domain was sterically oriented unfavorably towards the ER membrane, the MFY amino acids were not well conserved (YYK) and were buried in the putative peptide binding domain. In addition, Zhou *et al.* interpreted the crystal data to not favor higher order clustering of the IRE1α luminal domain. However, a GFP-tagged IRE1α demonstrably clusters during ER stress [54]. Interestingly, the human IRE1α luminal domain can functionally replace the Ire1p luminal domain [67,68]. Peptide studies for IRE1α, similar to the Gardner study, will be needed to help resolve the reported differences in the yeast and human systems.

## 5. The Regulatory Role of Kar2p/BiP

Does Ire1p/IRE1α, alone, directly sense misfolded protein stress and regulate the activation and deactivation of the UPR program? In light of how other misfolded stress sensing pathways include multiple regulatory players, usually in the form of chaperones, it would be surprising if Ire1p does it all.

As with other misfolded stress pathways, Ire1p/IRE1α binds to a chaperone, specifically Kar2p (the yeast homolog of BiP) and BiP. BiP (Kar2p) is the ER Hsp70, an ATPase that works in conjunction with co-chaperones (ERdj Hsp40 homologs and nucleotide exchange factors) to bind nascent and unfolded secretory proteins [69,70]. As unfolded secretory proteins accumulate, Kar2p/BiP binds these client proteins, preventing the unfolded clients from exiting the ER, and potentially poisoning later organelles of the secretory pathway [69,70,71,72]. Overexpression of BiP protects against UPR activation [73]. Conversely, decreased levels of BiP increase sensitivity to stress [74]. Thus, BiP appears to be the central node of an ER chaperone and homeostasis network [14,35,69,71,75]. BiP is upregulated 2–6-fold in UPR-adapted cells [76,77] as part of a global program to increase ER chaperone levels and improve the folding environment to substantially increase secretory protein synthesis and flux.

The Kar2p binding site has been mapped to Region V on the luminal domain of Ire1p (Figure 2) [35,49]. Kar2p/BiP releases from Ire1p/IREα as unfolded proteins accumulate [14,65,75,78,79]. BiP release was found to be coincident with the activation of Ire1p, both in terms of phosphorylation and dimerization [14,75,79,80]. In fact, BiP binds and releases from all three of the metazoan UPR stress sensors in a stress-dependent manner [14,16]. Release of BiP from the sensors is necessary for UPR activation [37]. Presumably, BiP/Kar2p sterically blocks IRE1α/Ire1p oligomerization. Several groups have speculated on a model in which Kar2p/BiP occupancy defines a threshold for Ire1p/IRE1α activation, such that upon titration of BiP by unfolded clients, BiP molecules associated with Ire1p/IRE1α will be released [14,49,60,65,75,81]. Unmasked/unobstructed Ire1p/IRE1α could now oligomerize and autophosphorylate [14,75].

There are some significant issues with a BiP titration model. First, Kar2p/BiP is one of the most abundant ER chaperones (~1-10 μM) [82,83]. Based on a ratio of levels of BiP and the Sec61 translocon, there are probably five to ten copies of BiP per active translocation channels for nascent proteins. One might predict that under only the most extreme stress conditions could the BiP pool become so occupied that the very low levels of Ire1p (~ three orders of magnitude less abundant than Kar2p [84]) or IRE1α would be unable to encounter free BiP [4,14]. Second, the UPR is not an all or none process. The UPR can occur over a spectrum of stressor levels in a dose-response-dependent manner [10,49]. IRE1α activation can occur at extremely low levels of pharmacologic stressors [10,85].

What happens if Kar2p/BiP binding of Ire1p is disrupted? Binding of Kar2p does not appear to be essential for regulating UPR activation of Ire1p [35,49]. Ire1p mutants that poorly bind Kar2p (if at all) [49,66] exhibit a low (three- to five-fold) constitutive level of UPR activation, distinct from a strong Tm or DTT induced stress (20–60-fold). In addition, *INO1* transcription is significantly constitutively upregulated in cells expressing an Ire1p mutant that binds little, if any, Kar2p (Ire1^bipless^) during unstressed conditions. Yet, the Ire1^bipless^ mutant can be induced 20-fold when stimulated with a misfolded protein stress [35,49]. The "bipless" mutant Ire1p does exhibit significantly greater sensitivities to ethanol and high temperature [35]. Exposure to ethanol induces a transient activation of wt Ire1p, but ΔV Ire1p (another Ire1p lacking the primary Kar2p binding domain, domain V) fails to attenuate *HAC1* splicing activity. It is unclear why Kar2p binding of Ire1p would be more important for heat or ethanol exposure. A wildly speculative possibility is that unlike DTT or Tm, heat and ethanol also impair general protein folding in the cytoplasm and the more global stress could affect a hypothetical cytoplasmic regulator of Ire1p activation, especially in the absence of Kar2p regulation. Alternatively, the unstructured linker domain of Ire1p could be sensitive to cytoplasmic misfolding conditions. In this scenario, the linker might undergo remodeling or misfold during biogenesis and enable oligomerization. Finally, it is notable that ethanol, heat, and inositol withdrawal could all impact lipid bilayer viscosity. Decreased viscosity could increase Ire1p collision rate and resulting activation. 

There is some controversy regarding whether the Ire1p mutants are truly free of Kar2p, as Kar2p is reported to be "sticky" in yeast (see next section) [49]. However, no obvious change in the amount of associated or disassociated Kar2p is observed upon misfolded protein stress-induced activation of the Ire1p mutant [35,49]. Interestingly, in mammalian cells, deletion of the BiP-binding domain of IRE1α results in a molecule that can still be induced to a highly active state by stress [86]. However, the unstressed IRE1α mutant is constitutively almost half as active as stress-induced wt IRE1α. Thus, BiP appears to play a more significant role in regulating IRE1α activation than in yeast.

It is unknown whether increasing concentrations of Ire1^bipless^ with peptide-binding mutations exhibit higher levels of constitutive activation. The prediction is that Ire1^bipless^ would tend to activate at much lower levels of overexpression than wild type, while the peptide-binding mutant would either be activated at levels similar to wt Ire1p regardless of unfolded peptide levels or would only become strongly constitutively active at much higher levels of mutant expression relative to the wild type. A more substantial mutant lacking both the Kar2p binding domain and a folded peptide-binding domain is not constitutively active and can barely be stimulated to activate, as measured by a β-galactosidase assay [37].

In yeast BiP-binding mutants, Ire1^bipless^ and similar ΔV or in mammalian cells, IRE1α 389, are not necessarily demonstrably free of Kar2p or BiP-binding [35,49,86]. In fact, even stressed wild type HA-tagged Ire1p only partially (~60%) releases Kar2p with the robust acute stress of DTT [49]. Thus, the regulatory role of Kar2p/BiP binding to and attenuating Ire1p/IRE1α requires further investigation. 

For wild type Ire1p, increased activation with increased expression either reflects (1) limiting amounts of Kar2p that can bind and inactivate Ire1p and/or (2) amounts of Kar2p that can buffer the unfolded secretory protein burden that could potentially bind to higher levels of Ire1p. While Kar2p and Ire1p have different binding specificities for peptides [36], Kar2p binding of unfolded proteins would likely sterically hinder peptide interactions with Ire1p.

In the peptide-binding model, Kar2p has been proposed to buffer the threshold for Ire1p activation, as well as the strength of the UPR [49]. Levels of DTT that barely activate a splicing reporter in wild type cells significantly activate Ire1^bipless^. An alternative model also proposes a buffering role for Kar2p binding to modulate Ire1p activity [65]. However, instead of a role for unfolded protein binding, the strength of Ire1p activation depends on the equilibrium between pools of Ire1p free inactive and oligomerized active pools. The transition between inactive and active was assumed to be highly cooperative. The dissociation constant of the equilibrium was such that if 70% of total Ire1p were released from Kar2p, half of that pool would be in the active state. In this model, the natural tendency of Kar2p-free Ire1p to oligomerize and the concentration of Kar2p bound and unbound Ire1p would be sufficient to regulate activation, independent of unfolded protein binding to Ire1p. The attractiveness of this model derives from its simplicity and the mechanistic similarity to other (i.e. HSF) misfolded protein stress sensing pathways that are strongly regulated by the binding and availability of a chaperone.

### 5.1. BiP Association—Stable or Transient?

Multiple studies report strong evidence that Kar2p strongly associates with Ire1p at steady state [14,35,79]. Binding of Ire1p/IRE1α by Kar2p/BiP has been described as "stable". The stability of the binding of BiP to IRE1α remains an open question. In light of the normal function of BiP, stable binding would prevent secretory proteins from *ever* folding. BiP must release from clients for a hydrophobic domain to be able to eventually be buried during the folding process. Bertolotti *et al.* inferred that the stability of binding occurred because of the *in vitro* release of BiP from IRE1α or PERK required addition of ATP [14]. However, BiP association with most clients depends on ATP hydrolysis to switch BiP from a low affinity to a high affinity form and release of hydrolyzed ADP from BiP to return to a low affinity form [72,87]. It is, therefore, not surprising that a purified BiP complex would not undergo significant release of a client. Otherwise one would anticipate that BiP-client complexes would be difficult to immunoprecipitate in the absence of crosslinkers. That said, most secretory proteins only transiently interact with BiP as an intermediate step in folding. The ability of Ire1p/IRE1α to continue to interact with Kar2p/BiP suggests, at the very least, that Ire1p/IRE1α contains a high affinity BiP binding site and/or an unfoldable domain. The Kar2p/BiP binding domain is unstructured in crystallography studies [38,60]. A potentially comparable situation occurs in antibody folding.

The immunoglobulin heavy chain contains an unfolded high affinity BiP binding site that only releases from BiP in the presence of the light chain binding partner [88]. BiP/Kar2p could bind IRE1α/Ire1p with an affinity comparable to that of its binding of the C_H_1 domain of immunoglobulin heavy chain (C_H_1), ~2.5–12 M [5,88]. This relatively tight association appears to be highly stable and BiP does not detectably cycle, only being released in the presence of the light chain, the partner for the heavy chain [88]. The exact mechanistic step that enables BiP to release from C_H_1 is not entirely clear [5]. Formation of an exposed disulfide bond in C_H_1 is essential for discontinuation of BiP binding. The unstructured regions [35,49] of Ire1p/IRE1α that associate with BiP lack cysteines, though there are additional cysteines in the luminal domain. However, a luminal domain mutated for cysteine residues remains functional, if a little more active in signaling compared to wild type [86]. If the stable binding model is correct, stress would somehow affect an IRE1α folding step to alter BiP affinity and this event would have to occur for multiple IRE1α molecules to collide and oligomerize.

What if Kar2p/BiP does cycle on and off Ire1p/IRE1α in cells? The experimental evidence for an absence of cycling is not iron clad. If BiP cycles on and off of Ire1p/IRE1α, how could the vast excess of free BiP play a role in a tunable system? One possibility is that little free BiP exists at steady state in cells. Surprisingly, there is no definitive study of this problem. It is unclear how many nascent proteins require BiP assistance for normal folding. Why would a cell keep little BiP in reserve for increased levels of unfolded proteins? This would place the cell at extreme risk of accumulation of aggregating misfolded proteins in the event of even a modest ER stress. A body of evidence suggests that the pool of BiP is only partially occupied at steady state. There is a substantial change in the size and fraction of BiP-containing complexes between homeostatic and acutely stressed cells [53,89,90,91]. This reflects increased binding of clients and a change in the size of client-containing complexes that could result from multiple chaperones binding to a single client, as can occur during stress [92].

If BiP does cycle, then one of the key mysteries of IRE1α regulation becomes simpler to resolve. No special regulatory steps would be required to compete BiP off of IRE1α. Accumulating misfolded secretory proteins would simply titrate the pool of free BiP, making it unavailable to bind and inactivate Ire1p/IRE1α. What about the pool of excess BiP of three orders of magnitude? The answer to this question depends on the extent of BiP occupancy at steady state. Biochemical and live cell assays of BiP occupancy suggest that a substantial pool of BiP is not client-bound or that client association is highly transient and clients are comparatively small [53,89,90,91]. During unfolded protein accumulation, clients can bind multiple chaperones, resulting in extremely large complexes and decreasing BiP availability.

If BiP does not cycle, why not and what would regulate BiP to release from IRE1α? Two major ways to prevent BiP from cycling would be to either (1) sterically hinder BiP access to the nucleotide exchange factor GRP170, which enables BiP to shift to a low affinity state and release clients and/or (2) post-translationally modify BiP bound to α. While IRE1α could sterically shield BiP from regulatory cofactors (*i.e*., GRP170 or ERdj3), it is unclear what would then make BiP more accessible during conditions of increasing levels of misfolded secretory proteins. 

Reports from the 1990s, as well as a recent study by Chambers *et al.*, have shown that BiP is posttranslationally modified in the metazoan ER lumen by ADP-ribosylation [93,94,95,96,97,98]. The modification converts BiP to a low affinity binding state and the modification is likely to be regulated by unidentified resident ER enzymes [93]. In stressed conditions, minimal ADP ribosylated BiP was detected in professional secretory cells [93,97] The current model of the processing of ADP-ribosylation is that only unoccupied BiP will be modified and inactivated [93]. During low client levels, ADP ribosylation would inactivate a pool of BiP, decreasing demands on ATP and decreasing retention of otherwise foldable secretory proteins [93]. As unfolded proteins accumulate constitutively de-ribosylated BiP becomes available to bind accumulating clients. Treatment with translational inhibitors or decreased translation in fasted rat pancreas leads to one third to half of BiP becoming ADP ribosylated [93]. Only when client levels decrease, will levels of ADP ribosylated BiP begin to increase again. Perhaps a decrease in available BiP to ribosylate could increase the probability that BiP bound to IRE1α could become deribosylated and lead to release from IRE1α. It would be interesting to determine whether a nonribosylatable form of BiP can bind or release from IRE1α. Also, it is unclear whether ADP ribosylation of BiP occurs in single cell organisms. 

The concentration of IRE1α/Ire1p in the ER membrane is an additional regulator of UPR activation. If most IRE1α is monomeric at steady state and expressed at very low levels, then BiP regulation gains further complexity, because Ire1p/IRE1α activation becomes an exceptionally low probability event. For any single IRE1α dimerization event, a BiP-free IRE1α molecule would need to diffuse throughout the tortuous network of ER tubule membranes (~20,000 μm^2^) [99] before potentially encountering another free IRE1α molecule. As free BiP increasingly binds to clients during unfolded protein accumulation, the probability of free IRE1α collisions begins to increase dramatically. Interestingly, Bertolotti *et al.* observed a substantial increase in BiP-bound IRE1α in cells overexpressing BiP [14]. Overexpressed BiP could decrease the probability that IRE1α will fail to encounter active BiP during normally stressful conditions.

Together, these data predict that a calculable threshold of clients is required for productive activation. Consistent with this model, increasing UPR sensors or decreasing BiP levels increases UPR activation, while increasing BiP will decrease stress sensitivity [35].

Another problem, how BiP associates with Ire1p/IRE1α, remains unresolved. The first question is whether BiP binds via its substrate-binding domain or some other region. The answer will impact the second problem of how BiP binding and release are regulated. Some studies suggest that BiP/Kar2p binds IRE1α/Ire1p via the BiP's substrate-binding domain. If correct, binding to Ire1p should resemble binding to other clients. Studies [16,35,68] have examined the effects of ATP hydrolysis mutants or in one case, client-binding mutants of Kar2p/BiP on regulated association with Ire1p/IRE1α [35]. The particular ATPase mutants are trapping mutants, as they cannot effectively release from clients, while substrate-binding mutants not only appear to not bind the UPR sensor, they cannot bind unfolded proteins, leading to exacerbated stress in yeast backgrounds lacking a wild type copy of *KAR2*. Thus, it is unclear whether substrateless Kar2p really cannot bind UPR sensors or if the constitutive stress resulting from the absence of functional Kar2p prevents the mutant from ever binding at the nonpermissive temperature. To resolve this, one could express a substrate-binding mutant of Kar2p (*kar2-133*) in *trans* in yeast endogenously expressing wild type Kar2p. At the permissive and nonpermissive temperatures, Ire1p could be immunoprecipitated from these cells and the amounts of wt and epitope tagged mutant Kar2p on Ire1p could be quantified. The endogenous Kar2p will maintain an unstressed environment, preventing global release of Kar2p from Ire1p. If mutant Kar2p binds Ire1p, the result would support an alternative model by Todd Corlett *et al.* [100] that Kar2p binds via a lobe on its ATPase domain to Ire1p.

The Ire1p-binding site for Kar2p has been mapped to a luminal region adjacent to the transmembrane domain [35]. This region is unstructured [38,60] in crystallography studies, similar to a BiP client, and suggests that BiP binding could be via the substrate-binding domain.

## 6. Ire1p Activation Independent of Unfolded Proteins: The Inositol Sensing Problem

Despite impressive efforts to characterize the role of BiP and peptide binding in the regulation of UPR activation, neither group of molecules appears to be the whole story. IRE1α can be activated by a small molecule targeted to its cytoplasmic domain [63]. Liu *et al.* [80], reported that replacing the luminal domain of Ire1p with a dimerizing leucine zipper motif (Maf or Jun) resulted in a constitutively weakly active Ire1p that could be substantially stimulated by ER stress (Tm) [80]. Whether peptide binding by the luminal domain is important during inositol depletion remains unclear. Constitutively dimeric Ire1p (with the luminal domain replaced with the Gcn4p bZIP domain) is inactive, but responsive to inositol depletion, which leads to clustering [37,59]. In addition, disruption of the peptide-binding domain (ΔIII) decreases sensitivity to DTT or Tm, but not inositol [59]. Wild type activation following inositol withdrawal is much slower than with unfolded protein stressors. Thus, there appears to be a way to stimulate Ire1p independent of the binding of unfolded proteins or release of BiP. Two recent studies further validate this phenomenon in relation to the atypical ER stress, inositol depletion.

Although Ire1p was first identified as an essential gene to prevent inositol auxotrophy [27,28,29,101], the connection between Ire1p and inositol regulation remains poorly understood. Ire1p's target mRNA, *HAC1*, is also required for yeast growth in inositol depletion conditions [27,32]. Ire1p activation by inositol depletion mirrors steps for unfolded protein activation Ire1p clustering [59], phosphorylation [52], splicing of *HAC1* [101,102], and an attenuation phase [49].

*IRE1* and *HAC1* are essential for yeast growth on inositol depletion media [27]. It remains a mystery why the UPR is required for growth in inositol depletion conditions. The relationship between Ire1p and the inositol pathway, which is regulated by *INO1* transcription, is unclear. Misfolded protein stresses significantly upregulate *INO1* transcription, as well as components of the *INO1* pathway [101]. Similarly, inositol depletion is accompanied by Ire1p activation and *HAC1* splicing [101]. Interestingly, Ire1p modification of *HAC1* is not necessarily required for growth in the absence of inositol [103]. Unspliced Hac1p has very low activity on UPR elements. It remains possible that Hac1p and Ire1p could have alternative functions for inositol depletion stress. Hac1p upregulates *INO2* and *INO4* levels [104] and reportedly enhances transcription of *INO1* [105]. UPR activation could also be a response to upregulation of *INO1* targets, which include ER membrane proteins.

In contrast to the peptide binding Ire1p activation model, inositol depletion does not require the Ire1p luminal domain. Promlek *et al.* reported that inositol depletion activates Ire1p lacking the peptide-binding domain [59]. In fact, the entire luminal domain of Ire1p can be replaced with the bZIP oligomerization domain of Gcn4p. The construct exhibits low constitutive activation, but can be stimulated substantially by inositol depletion [59]. Tm and DTT treatments can stimulate the same reporter, though activation is significantly slower than with wild type Ire1p [59]. Until recently, it has been unclear whether inositol depletion causes misfolded secretory proteins to accumulate in the ER. This does not appear to be the case. Lajoie *et al.* [89], found that inositol depletion is not associated with increased occupancy of Kar2p. Together, these data strongly suggest that *Ire1p can activate independently of unfolded peptide sensing.*

**Figure 3:**
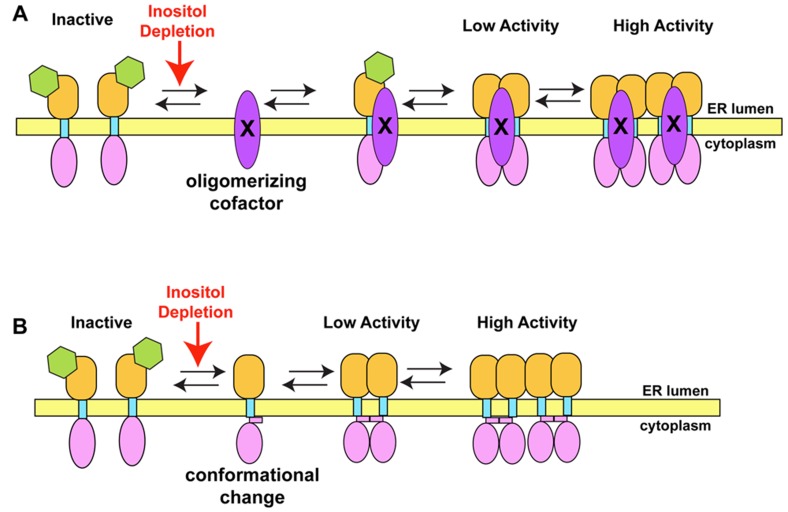
Alternative Ire1p activation Models. (**A**) Co-factor model. Upon inositol depletion, a membrane-associated or integral membrane protein accumulates in the ER membrane. The factor has affinity to the Ire1p cytoplasmic domain and enhances binding of pairs of Ire1p as Kar2p dissociates during normal binding and release or specifically binds and oligomerizes newly synthesized Kar2p-free Ire1p. (**B**) Direct sensing model. Ire1p detects changes in inositol levels and allosterically reorganizes or partially misfolds to increase affinity of the cytoplasmic domains leading to oligomerization and activation. Upon restoration of homeostasis Ire1p could resume the inactive conformation.

How inositol depletion and atypical stresses activate Ire1p remains a fundamental problem for the UPR field. Potential models include (1) a detector model in which a protein cofactor senses changes in lipids or metabolites and activates Ire1p through stimulated oligomerization and (2) a direct detection of a lipid metabolite or lipid by Ire1p that would induce a conformational change in the cytoplasmic domain, enabling oligomerization (Figure 3).

### 6.1. Ire1p or Protein Co-factor Detects Inositol Changes Model

Besides Ire1p luminal domain recognizing unfolded peptides, other Ire1p domains may recognize other types of substrates. For example, Contreras *et al.,* recently described the ability of a protein's transmembrane domain (TMD) to recognize sphingolipids [106]. In addition, the flavanol Quercetin [63] can bind the Ire1p cytoplasmic domain and potentiate Ire1p activation.

However, there is a conceptual problem with a cytoplasmic partner activating Ire1p. If the Ire1p luminal domain normally depends on peptide binding and release of Kar2p for maximal activation, it is unclear how the cytoplasm might trigger Kar2p release from the luminal domain, especially in the absence of obvious unfolded protein levels [89]. If Kar2p actually cycles on and off Ire1p, a cytoplasmic factor could conceivably forcibly bring two Ire1p molecules together in an orientation that would preclude rebinding of Kar2p. Alternatively, a co-factor could be an integral membrane protein with a luminal domain capable of stimulating Kar2p release from Ire1p. It would be interesting to determine whether, as with unfolded protein stress, increased Kar2p levels have any effect on regulation of Ire1p activation during inositol withdrawal.

### 6.2. How Might Ire1p Be Activated During Inositol Withdrawal or in the Absence of a Luminal Domain?

It is unknown what domain(s) of Ire1p are necessary for sensing inositol depletion and/or activation during inositol depletion. Thus, the cytoplasmic domain and/or a partner protein for the cytoplasmic domain are the most logical candidates. Identifying relevant regions of the cytoplasmic domain may prove difficult. Deletion of less than 10% of the cytoplasmic COOH-terminus of Ire1p renders the protein inactive [52,55]. Chimeras with human IRE1α domains may prove fruitful.

What about a cytoplasmic sensor of inositol levels and/or a regulator of Ire1p activation? Hetz and colleagues have described how mammalian cytoplasmic proteins can bind and regulate IRE1α activity [107,108,109]. In addition, Marcu *et al.,* described how Hsp90 binding to the cytoplasmic domain of IRE1α decreases the rate of IRE1α turnover [110]. It is unclear whether Hsp90 (Hsc82 or Hsp82) plays a similar role for Ire1p in yeast. The existence of cytoplasmic IRE1α co-factors establishes a precedent for IRE1 function and regulation independent of unfolded secretory protein levels in the ER lumen. In addition, Marcu *et al.*, reported binding of IRE1α by GRP94, the luminal Hsp90 homolog [110]. As HSF1 binds Hsp90, a role for GRP94 certainly remains a possibility. However, yeast lacks a luminal Hsp90. Yet, the human IRE1α luminal domain can replace the yeast Ire1p luminal domain and retain robust stress-inducible activation with only a trace of unstressed constitutive activation [80]. Thus, GRP94 does not appear to be critical for negative regulation of IRE1α.

How might a cytoplasmic co-factor regulate Ire1p during inositol depletion? Given the importance of oligomerization for Ire1p activation, a cytoplasmic clustering factor for Ire1p makes sense. The molecule would require some sort of activation to become an Ire1p partner and binding to Ire1p could be transient to permit downregulation of Ire1p activity. Coupled with the low abundance of Ire1p, such a molecule could be biochemically challenging to detect. Furthermore, if the clustering factor were an essential protein with other inositol-related functions, it would be difficult to pick such a needle out of a haystack.

How would an alternative stress stimulate release of BiP from Ire1p? If the stress perturbed BiP function, such as calcium depletion [111], this could lead to Kar2p release. Another possibility is that the hypothetical regulatory molecule could bind one Ire1p and in rare, but still with sufficiently frequent probability, cases capture another Ire1p shortly after a Kar2p dissociates. Once bound, the co-factor would need to facilitate Ire1p activation, perhaps by binding to a site similar in function to the quercetin-binding site on Ire1p that activate Ire1p RNase activity [63]. The quercetin binding site is not a candidate, as Promlek *et al.*, mutated those residues and observed no difference in inositol depletion mediated activation [59]. The slow accumulation of activated Ire1p molecules would make the process more linear, and thus less acute, than a more familiar misfolded protein stress. Such a model would be consistent with studies to date and could serve as a template for other ways to activate or regulate the UPR.

## 7. Ire1p Inactivation

Just as it has been challenging to understand how Ire1p/IRE1α can be activated by distinct stresses and to varying degrees, attenuation and deactivation of the UPR represent similarly difficult problems. First, there is the general matter of the steps of deactivation. Does it proceed by gradual dissociation of dimers from clusters followed by monomerization? Do individual monomers break off of clusters? Are peptides competed off of luminal domains by increasing levels of Kar2p/BiP? Does attenuation correlate directly with increased Kar2p/BiP levels? If so, is there a threshold level of Kar2p/BiP for attenuation? Does overall BiP occupancy decrease detectably in Ire1p attenuating cells?

Perhaps the most puzzling question is, how does Ire1p/IRE1α attenuate/deactivate in cells in the continued presence of an active stressor [19,44]? Lin *et al.*, reported that human cell lines attenuated *Xbp1* splicing after 8 h of exposure to Tm, that the Tm did not lose potency, and other arms of the UPR (especially PERK) continued to remain active [44]. If the PERK luminal domain can substitute for the IRE1α luminal domain [14,80], then attenuation is likely to reflect inactivation at the cytoplasmic domain of IRE1α. The groups of Peter Walter and Maho Niwa reported that increasing levels of autophosphorylation are believed to enhance repulsion of Ire1p monomers from clusters [19,20], and this repulsion could drive inactivation of the UPR. Newly upregulated levels of Kar2p/BiP could be sufficiently available to bind unfolded secretory proteins, competing them away from Ire1p oligomers, and also directly binding free Ire1p monomers. Interestingly, studies by our group showed little change in Kar2p-sfGFP mobility during the time period when the UPR attenuates (4 h), whereas Kar2p mobility fully recovered by 16 h [89]. At 4 h of exposure to a robust dose of Tm, splicing reporter fluorescence had increased maximally [89]. A phosphatase could then potentially reset the status of the Ire1p monomers to a deactivated state [112,113]. It is likely that FRAP diffusion measurements are not sufficiently sensitive to detect small changes in Kar2p availability, *i.e.*, an increase of 10% of unbound Kar2p molecules. However, if relatively small changes in Kar2p BiP availability govern Ire1p inactivation, it becomes a little more difficult to understand how the UPR can be tuned to a wide range of levels of stressors. If peptide binding were the key regulator, then increasing levels of chaperones entering the ER would titrate the available pool of unfolded proteins due to higher levels of chaperones and increased competition by UPR upregulated mRNAs for translocation channels. That is, the misfolded protein mRNA becomes a smaller percentage of total mRNA and comparatively less misfolded secretory protein would be produced. How attenuation would work for the constructs that have luminal domains replaced with bZIP dimerization domains that cannot bind peptides or BiP is unclear. While the constructs do not appear to be detrimental in the way that kinase inactive Ire1p mutants impair cell recovery during stress [19,20,80], it has not been reported whether the constructs attenuate and if so, when. If a cytoplasmic regulator enhances Ire1p oligomerization during inositol depletion, restoration of homeostasis could result in release/turnover of the regulator, followed by dispersal of Ire1p.

Once homeostasis (a manageable level of unfolded proteins) is restored, Ire1p/IRE1α would disassociate as increased levels of Kar2p competed for unfolded secretory peptides. The increasingly extensive phosphorylation of Ire1p during activation [52] has been proposed as a mechanism for Ire1p disassociation. The negative charges could repulse Ire1p molecules away from each other and one or more phosphatases (Dcr2p and/or Ptc2p) would return the Ire1p molecules to a resting and activatable state [112,113].

## 8. Conclusion

The emerging picture of Ire1p regulation is that peptide binding to the MHC1-like groove can enhance the rate of Ire1p activation and its splicing activity in yeast. Yet, peptide binding does not appear to be essential for Ire1p function. UPR activating conditions exist where no apparent increase in unfolded protein levels occur.

It is striking how many similarities and differences there are between Ire1p/IRE1α and other unfolded protein response pathways. Ire1p, σ32, and HSF all interact with chaperones to help regulate activity. HSF and σ32 directly regulate transcription of the chaperone that binds them, while Ire1p/IRE1α regulates processing of the transcription factor that upregulates Kar2p/BiP. HSF, σ32, and IRE1α are all regulated directly by chaperone binding and release, apparently through changing levels of unfolded proteins that can interact with the regulatory chaperone. Ire1p's regulation remains more controversial. Whether Kar2p binding is very important requires resolving, as does the question whether Ire1p mutants are truly free of associated Kar2p. The peptide-binding model has considerable supporting evidence, though the UPR can clearly be activated without the Ire1p luminal domain. It is also surprising that IRE1α could be so radically different in mechanism compared to Ire1p. It remains to be determined whether IRE1α can bind unfolded peptides, but the crystal structure either reflects a closed form of the peptide-binding domain [36] or could represent the evolutionary remnants of a mechanism unfavorable for metazoans.

Despite considerable sequence conservation in IRE1 homologs, the UPR, even in single celled fungi, can exhibit surprising diversity. Recently the laboratory of Jonathan Weissman noted that *Schizosaccharomyces pombe* expresses Ire1, but lacks *HAC1* in its genome. Importantly, strong genetic links between Ire1p and the *S. pombe* UDP-glucose-glycoprotein glucosyltransferase and calnexin were identified [114]. Similar links were not detected between *IRE1* and the *S. cerevisiae* homologs. Thus, the UPR appears to be malleable. The capacity for RIDD remains to be explored in fungi.

How Ire1p is activated is likely to be important for the resulting UPR. Thibault *et al.*, recently reported that deletions of different important ER genes led to comparable upregulation of the UPR transcriptional reporter assay, but resulted in gene regulation profiles different from each other and a standard DTT stress [64] This could reflect activation of additional stress pathways, such as the Heat Shock pathway [115]. Alternatively, just as acute stress and inositol depletion can both result in comparable levels of UPR activation, but at different rates, how stresses activate the UPR may be critical for predicting cellular outcomes.

The complexity of UPR regulation in yeast hints at even richer possibilities in human cells. With five sensors, (IRE1α and β, PERK, and ATF6α and β), evidence of multiple cytoplasmic regulators of UPR sensors [107,108,109,110], differences in sensitivities to distinct stresses [116], and recent studies hinting at the abilities of novel sensing mechanisms (*i.e.*, ATF6 appears to detect transmembrane domains [117]), there is little doubt that the human UPR will hold many surprises. Understanding this complexity will become increasingly important as new studies identify UPR regulatory roles beyond the management of unfolded protein levels [22]. Increasing connections are being recognized between dysregulated UPR and human disease. Alternative UPR regulatory modes are providing new therapeutic targets at additional sites on the sensors and modulation of protein co-factors.
